# Supplementary material for: Polyketide synthase gene clusters in the ericoid mycorrhizal fungus Oidiodendron maius: genome-wide prediction, expression profiling, and functional characterization of knockout mutants
Source: Front Plant Sci. 2026 Sep 16;17:1924430. doi: 10.3389/fpls.2026.1924430 (PMC13623903; doi:10.3389/fpls.2026.1924430)
Supplement: Supplementary file 1 [file SupplementaryFile1.zip › Supplementary Materials/Supplementary Folder 1.zip!/Oidma1_AssemblyScaffolds_Repeatmasked/knownclusterblast/cluster51/jgi.p_Oidma1_173217_mibig_hits.html]

| MIBiG Protein | Description | MIBiG Cluster | MiBiG Product | % ID | % Coverage | BLAST Score | E-value |
| --- | --- | --- | --- | --- | --- | --- | --- |
| ADY16687.1 | TqaC | BGC0001142 | Tryptoquialanine\_biosynthetic\_gene\_cluster | 33 | 97.5535168196 | 149 | 6.4e-36 |
| BAJ09787.1 | dehydrogenase | BGC0000146 | Solanapyrone\_biosynthetic\_gene\_cluster | 36 | 80.7339449541 | 129 | 8.9e-30 |
| EDY47129.1 | conserved\_hypothetical\_protein | BGC0000845 | Clavulanic\_acid\_biosynthetic\_gene\_cluster | 27 | 48.3180428135 | 51 | 1.8e-06 |
